# Hematocrit and 28-day mortality in mechanically ventilated septic patients: a prospective observational study

**DOI:** 10.3389/fmed.2026.1890758

**Published:** 2026-08-26

**Authors:** Lianghai Cao, Youlian Zhou, Pengcheng Shen, Hui Cui, Jin Tang, Min Xiao, Ping Fang, Guilan Ding, Jun Qiu

**Affiliations:** 1 Chengdu Integrated TCM and Western Medicine Hospital, Chengdu, Sichuan, China; 2 The First People’s Hospital of Yibin, Yibin, Sichuan, China; 3 West China Hospital, Sichuan University, Sichuan University West China Hospital Guang'an Hospital, Guang’an People’s Hospital, Guangan, Sichuan, China; 4 Affiliated Hospital of North Sichuan Medical College, Nanchong, Sichuan, China; 5 The Second People’s Hospital of Neijiang, Neijiang, Sichuan, China; 6 Dazhou Central Hospital, Dazhou, Sichuan, China

**Keywords:** hematocrit, mechanical ventilation, mortality, prospective observational study, sepsis

## Abstract

**Background:**

Sepsis is a condition that can lead to severe complications and is estimated to affect 300 million people annually. Patients requiring mechanical ventilation represent a particularly critical subgroup with high mortality. While hematocrit (HCT) has been identified as a prognostic marker in general sepsis populations, its specific role in mechanically ventilated patients remains unexplored. The objective of this study is to examine the relationship between HCT levels and 28-day mortality in this vulnerable patient cohort.

**Methods:**

In this prospective observational study, an exploratory candidate cutoff for HCT was empirically derived by maximizing the Youden index from the ROC curve to predict 28-day mortality. The cohort was then stratified into low-HCT and high-HCT groups, utilizing the aforementioned candidate threshold. To assess the stability of this data-driven cutoff, an internal bootstrap validation procedure was performed. Subsequently, the association between HCT stratification and 28-day mortality was evaluated using a Cox proportional hazards regression model, adjusting for prespecified covariates.

**Results:**

Among the 233 patients included in the study, 40 (17.2%) died within 28 days. Patients were stratified into two groups, low HCT (<26.65%) and high HCT (≥26.65%), based on the exploratory HCT cutoff of 26.65%. In the multivariable Cox proportional hazards model, patients in the low HCT group exhibited a significantly elevated risk of 28-day mortality compared to those in the high HCT group (adjusted hazard ratio [HR] = 2.16, 95% CI: 1.04–4.52; *p* < 0.05).

**Conclusion:**

In this multicenter study, an exploratory HCT cutoff of 26.65% was identified, and it was observed that low HCT was associated with a 2.16-fold increased risk of 28-day mortality in mechanically ventilated sepsis patients. However, the discriminative ability of HCT was modest (AUC ≈ 0.537), suggesting limited predictive utility as a standalone test. Given its low cost and routine availability, HCT may still hold value as an adjunctive variable in risk stratification. However, further investigation is necessary to validate its incremental prognostic value and determine its optimal integration with other established predictors in larger, well-powered cohorts.

## Introduction

Sepsis, defined as a life-threatening organ dysfunction that results from dysregulated host responses to infection, poses a significant challenge in clinical practice. A recent Global Burden of Diseases report documented 48.9 million global sepsis cases in 2017 with a case mortality rate of 22.5%, constituting nearly 20% of all global deaths (1–3). Patients with sepsis who also develop acute respiratory failure (ARF), necessitating mechanical ventilation, represent a distinctive and intricate clinical subcategory (4–6). Prior studies have indicated that approximately 20% of patients with sepsis develop respiratory failure, a condition that may be associated with uncontrolled inflammation, fluid resuscitation, and abnormal clotting (7). The clinical complexity of this subgroup underscores the necessity for robust and accessible biomarkers to enhance risk stratification and guide therapy.

In patients with sepsis, the systemic inflammatory cascade has been demonstrated to increase capillary permeability, promote fluid extravasation, and induce fluctuations in hematocrit (HCT) (8). Low HCT, indicative of diminished oxygen-carrying capacity, has been correlated with elevated mortality rates in sepsis populations (9). However, in patients requiring mechanical ventilation (MV), the underlying pathophysiological context is considerably more intricate. Although mechanical ventilation (MV) is an effective life-saving intervention, it can also amplify systemic inflammation and lung injury through ventilator-induced lung injury (VILI) mechanisms (5). This phenomenon has been conceptualized as a “two-hit” pathophysiology, whereby the initial septic insult (“first hit”) primes the host immune system and renders lung tissue vulnerable, and the subsequent application of MV (“second hit”) further exacerbates preexisting injury. Experimental evidence demonstrates that MV, which may be relatively innocuous in healthy lungs, markedly amplifies inflammatory responses in the septic state (10). This amplified inflammatory milieu may, in turn, exacerbate endothelial injury and dysregulate fluid homeostasis, thereby further perturbing HCT beyond the effects of sepsis alone.

A recently developed LASSO-based prediction model identified HCT as one of ten key variables most strongly associated with sepsis-related ICU mortality (11). However, the specific behavior and prognostic value of HCT in the subset of septic patients receiving MV remain poorly characterized, warranting further investigation. Accordingly, the objective of this study was to evaluate the association between HCT levels and 28-day mortality in mechanically ventilated septic patients, with the goal of informing risk stratification in this vulnerable population.

## Methods

### Participants

**Figure 1:**
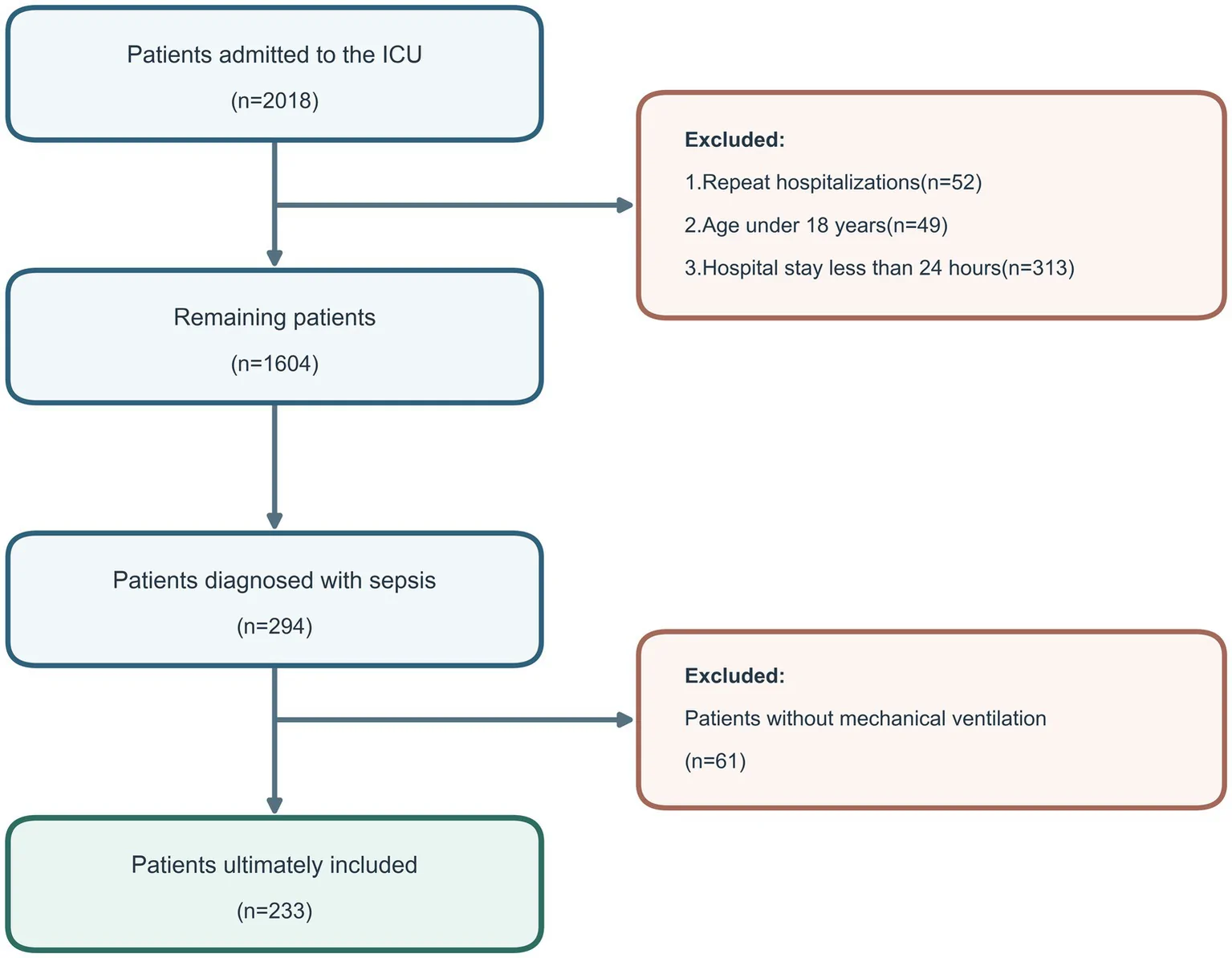
Flowchart of inclusion and exclusion criteria.

This multicenter, prospective observational study was conducted between October 10, 2017, and January 9, 2018, across the intensive care units (ICUs) of seven tertiary hospitals in Sichuan Province, China. The study protocol received approval from the Ethics Committee of the Affiliated Hospital of North Sichuan Medical College (No. 2017ER(R)022) and the respective committees of all participating sites, and was registered with the China Clinical Trial Center (#ChiCTR-EOC-17012679), which operates under guidelines that are consistent with the core principles of the Declaration of Helsinki. The study population comprised patients admitted to the intensive care unit (ICU) with a diagnosis of sepsis according to the Sepsis-3 criteria (12), including both patients with and without septic shock. Patients with repeat ICU admissions, those below the age of 18, a length of stay less than 24 h, and those who did not receive mechanical ventilation were excluded from the study. The patient selection process is delineated in Figure 1.

### Data collection

The extraction of data from patient records and electronic databases was carried out by trained researchers at each site, under the supervision of a local coordinator. The identification of patients and the recording of data on standardized paper case report forms (CRFs) were conducted prospectively in real time. The exposure (HCT) and outcome (28-day mortality) were predefined in the study protocol. All CRFs were subsequently submitted to the central coordinating center (Affiliated Hospital of North Sichuan Medical College) for processing. Subsequently, a rigorous quality control procedure was implemented, which included an audit by specialist monitors to ensure data integrity and resolve discrepancies. Following a comprehensive audit and data set cleansing, the data was entered into a customized EpiData (v3.0) database through a dual-entry process. All participants in the study provided voluntary written informed consent at the time of ICU admission.

### Outcome and variables

The primary research variable was hematocrit (HCT), and the values included were the highest recorded after patients were admitted to the intensive care unit. The primary outcome was 28-day mortality. The following covariates were considered in the analysis: demographic characteristics (age, sex); comorbidities (chronic obstructive pulmonary disease, coronary heart disease, hypertension, diabetes, chronic kidney disease, malignancy); vital signs (body temperature, heart rate, respiratory rate, mean arterial pressure); and laboratory parameters (proportion of neutrophils, platelet count, procalcitonin, total bilirubin, albumin, creatinine, potassium, sodium, lactate). Vital signs and laboratory parameters were documented as the most abnormal values observed post-ICU admission. The additional treatment-related variables encompass the use of vasopressors and continuous renal replacement therapy. The severity of illness was assessed using the Sequential Organ Failure Assessment (SOFA) scores. The SOFA scores were calculated on the second day following diagnosis using the worst values of the relevant clinical indicators during that period. The present time point was selected because it reflects the peak severity of organ dysfunction after initial resuscitation. This provides more informative prognostication for this cohort than the admission SOFA score.

### Statistical analysis

An exploratory cut-off point for HCT in predicting 28-day mortality was empirically selected by maximizing the Youden index on the receiver operating characteristic (ROC) curve. In order to assess the stability of this candidate threshold and to guard against potential overfitting bias, an internal validation was performed using bootstrap resampling. Specifically, 1,000 bootstrap samples were generated by resampling the original dataset with replacement, with each sample size maintained at the original cohort size. For each bootstrap replicate, the Youden index was re-maximized to re-estimate a new cutoff. The robustness of the initially identified exploratory cutoff was evaluated by examining the distribution (median, quartiles, and standard deviation) of the 1,000 resulting cutoff values. Patients were then categorized into two groups based on this cut-off: a low HCT group, defined as below the cut-off, and a high HCT group, defined as above or equal to the cut-off. Continuous variables were presented as median with interquartile range (IQR) and compared using the Mann–Whitney U test. Categorical variables were expressed as frequency with percentage and compared using the Chi-square test or Fisher’s exact test, as appropriate. The survival curves were plotted using the Kaplan–Meier method and subsequently compared with the log-rank test. To assess the independent association between HCT and 28-day mortality, a multivariable Cox proportional hazards model was developed. Candidate variables were preselected based on clinical relevance from prior literature (13, 14) and univariate associations at a threshold of *p* < 0.01. The presence of multicollinearity among these preselected covariates was assessed using the variance inflation factor (VIF). Given the limited number of events (*n* = 40) relative to the five covariates included, the events-per-variable (EPV) ratio was approximately 8, indicating a potential risk of overfitting. To quantify this uncertainty and evaluate the stability of the model, an internal bootstrap validation was performed, with 1,000 resamples. In each replicate, the full Cox model was refitted. The distribution of the 1,000 bootstrap-derived hazard ratios for HCT was summarized to assess the robustness of the point estimate. Statistical significance was determined by a two-sided *p*-value < 0.05. All analyses were performed using R software (version 4.3.1).

## Results

### Population and baseline characteristics

**Figure 2:**
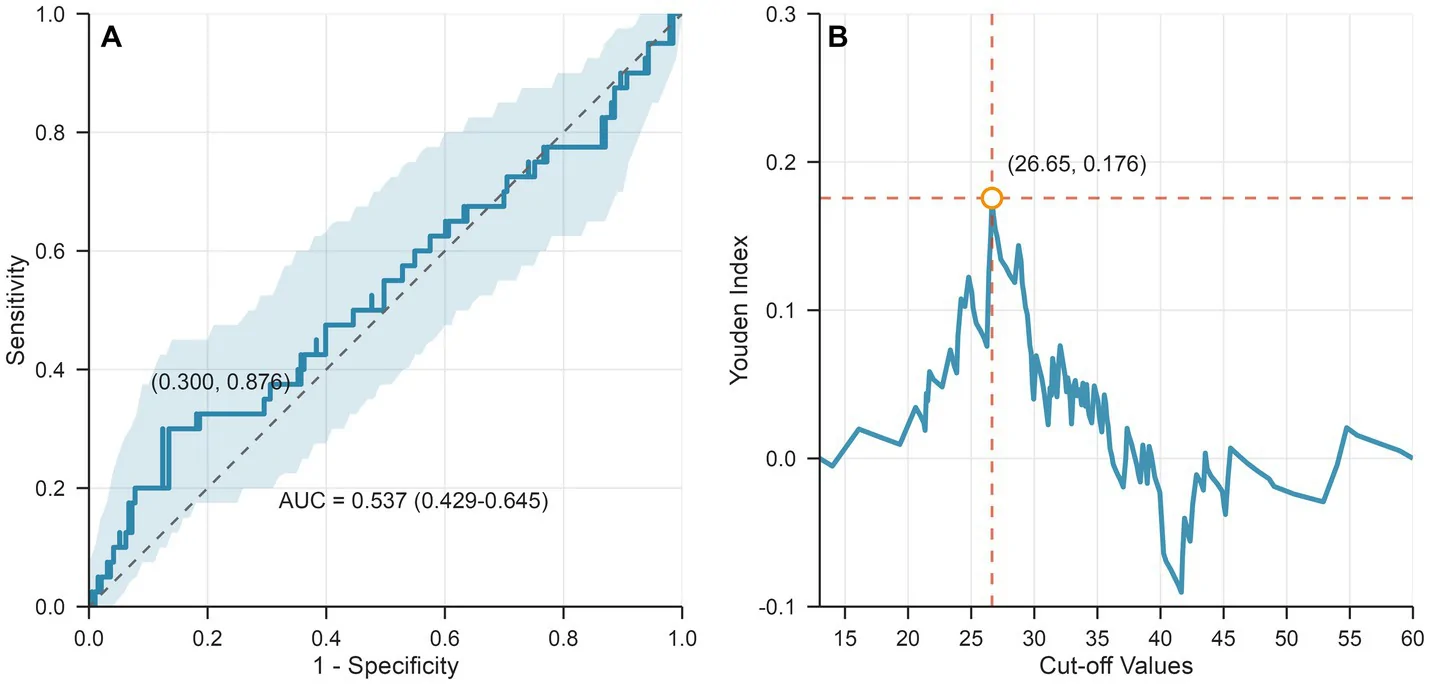
**(A)** Receiver operating characteristic (ROC) curve for HCT. **(B)** The relationship between HCT and Youden Index.

Based on the Youden index applied to the current dataset, an exploratory candidate cutoff for HCT was identified at 26.65%, yielding a maximum Youden index of 0.176 (Figure 2). To evaluate the internal stability of this empirically derived threshold, we performed a bootstrap internal validation with 1,000 resampling iterations. The distribution of the re-estimated cutoffs across these bootstrap samples showed a median of 28.75%, an interquartile range of 26.65–35.60%, and a standard deviation of 6.20%. Notably, the originally identified exploratory cutoff of 26.65% coincided exactly with the first quartile of this bootstrap distribution, indicating that this candidate value lay within the interquartile range of the resampled estimates and exhibited reasonable internal consistency in this cohort. Patients were then stratified into low-HCT (<26.65%) and high-HCT (≥26.65%) groups. The final analysis included 233 patients, of whom 40 (17.2%) died within 28 days. Baseline characteristics of the study population are summarized in Table 1. A comparative analysis of survivors and non-survivors yielded significant disparities in demographic characteristics, comorbidities, vital signs, laboratory parameters, interventions, and clinical scores. Specifically, compared with survivors, non-survivors were significantly older and had higher sodium levels, lactate levels, and SOFA scores, but lower arterial pressure and platelet counts (all *p* < 0.05). Furthermore, a higher prevalence of malignant neoplasms, the use of vasopressor agents, and low hematocrit levels was observed in non-survivors. It is noteworthy that no significant differences were observed in Hemoglobin between the two groups.

**Table 1:** Baseline characteristics of mechanically ventilated septic patients.

Characteristics	Total	Survivors	Non-survivors	*p* value
	(*N* = 233)	(*N* = 193)	(*N* = 40)	
Demographics				
Age (years)	68.0 (58.0, 76.0)	67.0 (55.0, 75.0)	72.5 (65.2, 82.5)	0.011
Gender, *N* (%)				0.147
Male	161 (69.1%)	129 (66.8%)	32 (80.0%)	
Female	72 (30.9%)	64 (33.2%)	8 (20.0%)	
Comorbidities				
COPD, *N* (%)				0.592
No	205 (88.0%)	171 (88.6%)	34 (85.0%)	
Yes	28 (12.0%)	22 (11.4%)	6 (15.0%)	
CHD, *N* (%)				0.129
No	201 (86.3%)	170 (88.1%)	31 (77.5%)	
Yes	32 (13.7%)	23 (11.9%)	9 (22.5%)	
Hypertension, *N* (%)				0.226
No	156 (67.0%)	133 (68.9%)	23 (57.5%)	
Yes	77 (33.0%)	60 (31.1%)	17 (42.5%)	
Diabetes, *N* (%)				0.852
No	186 (79.8%)	155 (80.3%)	31 (77.5%)	
Yes	47 (20.2%)	38 (19.7%)	9 (22.5%)	
CKD, *N* (%)				0.407
No	222 (95.3%)	185 (95.9%)	37 (92.5%)	
Yes	11 (4.7%)	8 (4.1%)	3 (7.5%)	
Malignant cancer, *N* (%)				0.018
No	219 (94.0%)	185 (95.9%)	34 (85.0%)	
Yes	14 (6.0%)	8 (4.1%)	6 (15.0%)	
Vital signs				
Temperature (°C)	38.0 (37.3, 38.6)	38.0 (37.3, 38.7)	37.6 (37.2, 38.3)	0.158
Heart rate (bpm)	130 (116, 147)	130 (117, 148)	129 (113, 142)	0.769
Respiratory rate (t/min)	25.0 (21.0, 30.0)	25.0 (22.0, 30.0)	24.0 (20.0, 30.0)	0.286
MAP (mmHg)	66.0 (58.7, 72.7)	66.7 (60.0, 73.3)	62.0 (55.4, 69.7)	0.007
Laboratory parameters				
HGB (g/L)	84.0 (70.0, 105)	84.0 (70.0, 105)	84.0 (70.2, 104)	0.744
NEUT (%)	92.3 (88.4, 94.8)	92.5 (88.8, 94.9)	91.4 (86.4, 94.3)	0.160
PLT (10^^9^/L)	80.0 (46.0, 141)	88.0 (47.0,147)	64.5 (20.8, 104)	0.025
PCT (ng/mL)	19.0 (6.29, 55.9)	18.2 (5.90, 54.8)	25.5 (12.0, 72.0)	0.177
TBIL (umol/L)	14.6 (9.80, 27.6)	14.6 (9.80,27.7)	14.6 (9.98, 25.4)	0.945
ALB (g/L)	25.3 (21.9, 28.7)	25.2 (21.8,28.4)	25.8 (22.5, 29.5)	0.429
CREA (umol/L)	140 (88.5, 252)	129 (87.2, 241)	197 (111, 279)	0.062
K (mmol/L)	4.64 (4.14, 5.18)	4.60 (4.10, 5.14)	4.79 (4.27, 5.31)	0.269
Na (mmol/L)	144 (140, 148)	143 (140, 148)	147 (142, 150)	0.019
Lac (mmol/L)	4.40 (2.99, 7.64)	4.30 (2.60, 7.15)	7.32 (4.00, 11.9)	<0.001
Interventions				
Vasopressors, *N* (%)				<0.001
No	90 (38.6%)	86 (44.6%)	4 (10.0%)	
Yes	143 (61.4%)	107 (55.4%)	36 (90.0%)	
CRRT, *N* (%)				0.757
No	213 (91.4%)	177 (91.7%)	36 (90.0%)	
Yes	20 (8.6%)	16 (8.3%)	4 (10.0%)	
SOFA	10.0 (7.00, 13.0)	10.0 (7.00, 12.0)	11.0 (7.75, 16.0)	0.028
HCT, *N* (%)				0.011
High	197 (84.5%)	169 (87.6%)	28 (70.0%)	
Low	36 (15.5%)	24 (12.4%)	12 (30.0%)	

COPD, chronic obstructive pulmonary disease; CHD, coronary heart disease; CKD, chronic kidney disease; MAP, mean arterial pressure; HGB, hemoglobin; NEUT, neutrophil; PLT, platelet; PCT, procalcitonin; TBIL, total bilirubin; ALB, albumin; CREA, creatinine; HCT, hematocrit.

### Kaplan–Meier survival curve analysis

**Figure 3:**
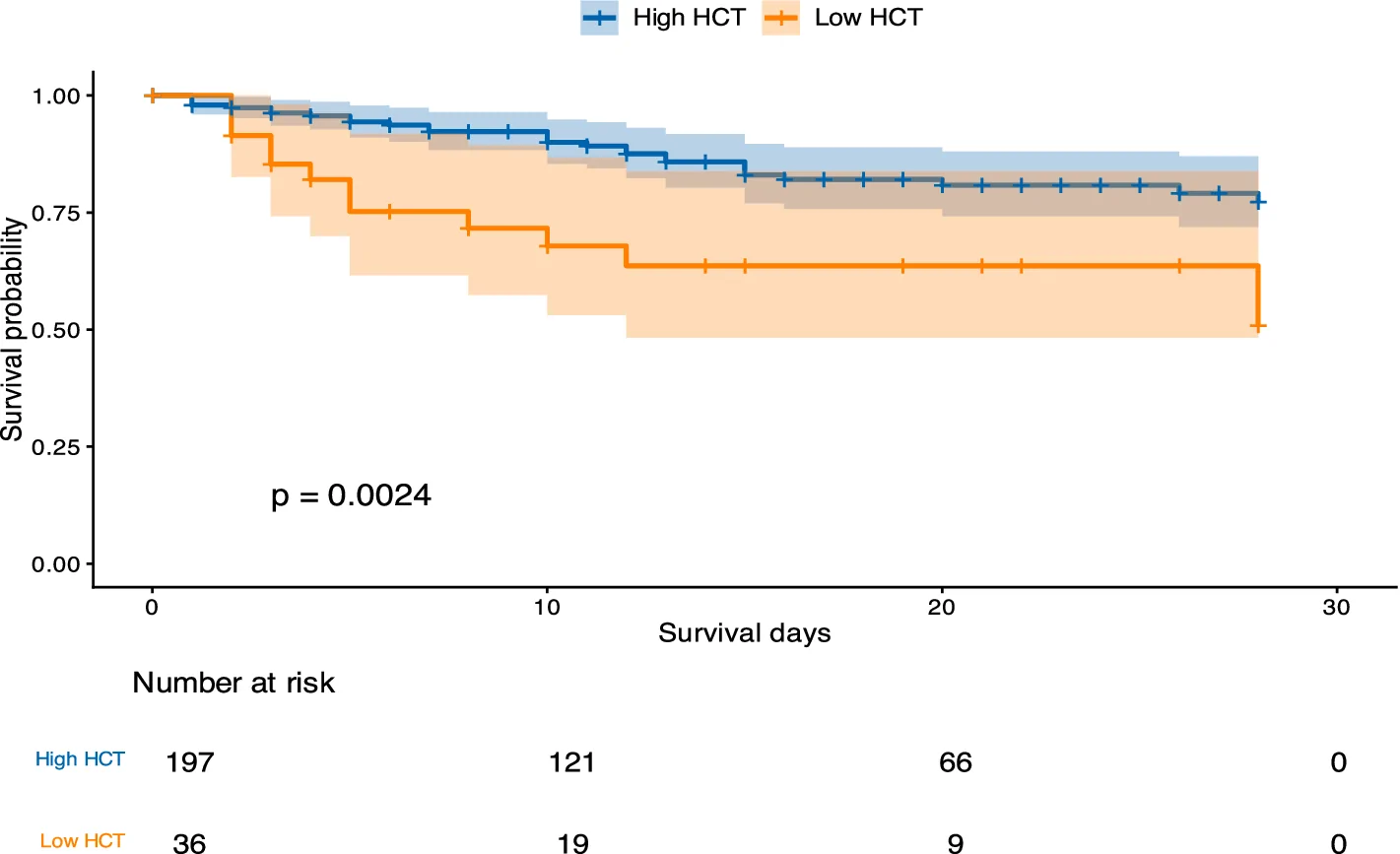
Survival curves for 28-day mortality according to HCT group.

The Kaplan–Meier analysis revealed a significantly lower 28-day survival probability in the low-HCT group (*n* = 36) compared to the high-HCT group (*n* = 197) (log-rank test, *p* < 0.05; Figure 3), indicating that low hematocrit was associated with increased mortality.

### Prognostic value of hematocrit in sepsis with mechanical ventilation

In univariate Cox regression analysis, significant predictors of 28-day mortality included age, malignancy, mean arterial pressure, neutrophil percentage, platelet count, sodium, lactate, vasopressor use, SOFA score, and HCT (all *p* < 0.05). The following five covariates were subsequently entered into the multivariable model based on *p* < 0.01 and clinical relevance: mean arterial pressure, lactate, vasopressor use, SOFA score, and HCT. The values of the variance inflation factor were all below 5, indicating no substantial multicollinearity (see Appendix 1). In comparison to the high HCT group, the low HCT group exhibited an adjusted hazard ratio of 2.16 (95% CI: 1.04–4.52; *p* < 0.05) (Table 2). Given the modest event count (*n* = 40, EPV ≈ 8), the estimate is inherently uncertain. Bootstrap internal validation (1,000 replicates) revealed substantial variability in HCT hazard ratios (median: 2.44; interquartile range [IQR]: 1.78–3.36; standard deviation [SD]: 1.84), with a 95% confidence interval of 0.858–6.579 that crossed unity. These findings suggest that the observed association may be sample-dependent, and thus the result should be interpreted as exploratory, pending independent validation.

**Table 2:** Univariate and multivariable analyses of predictors for 28-day mortality.

Variable	Category	Total (*N* = 233)	Univariable HR (95% CI), *p* value	Multivariable HR (95% CI), *p* value
Age	Mean±SD	65.3 ± 15.6	1.03 (1.00–1.05, *p* = 0.020)	
Gender	Male	161 (69.1%)		
	Female	72 (30.9%)	0.49 (0.22–1.06, *p* = 0.070)	
COPD	No	205 (88.0%)		
	Yes	28 (12.0%)	1.70 (0.71–4.06, *p* = 0.235)	
CHD	No	201 (86.3%)		
	Yes	32 (13.7%)	1.90 (0.91–4.00, *p* = 0.089)	
HBP	No	156 (67.0%)		
	Yes	77 (33.0%)	1.48 (0.79–2.77, *p* = 0.222)	
Diabetes	No	186 (79.8%)		
	Yes	47 (20.2%)	1.12 (0.53–2.36, *p* = 0.760)	
CKD	No	222 (95.3%)		
	Yes	11 (4.7%)	1.53 (0.47–4.96, *p* = 0.480)	
MA	No	219 (94.0%)		
	Yes	14 (6.0%)	2.40 (1.01–5.71, *p* = 0.049)	
T	Mean ± SD	38.0 ± 1.0	0.74 (0.51–1.06, *p* = 0.095)	
HR	Mean ± SD	129.9 ± 24.8	1.00 (0.99–1.02, *p* = 0.601)	
R	Mean ± SD	26.1 ± 8.0	0.98 (0.94–1.02, *p* = 0.302)	
MAP	Mean ± SD	65.3 ± 17.1	0.97 (0.95–0.98, *p* < 0.001)	0.98 (0.96–1.00, *p* = 0.032)
HGB	Mean ± SD	87.9 ± 26.1	1.01 (0.99–1.02, *p* = 0.367)	
NEUT	Mean±SD	90.1 ± 10.0	0.98 (0.96–1.00, *p* = 0.028)	
PLT	Mean ± SD	101.0 ± 77.2	0.99 (0.99–1.00, *p* = 0.027)	
PCT	Mean ± SD	35.4 ± 38.0	1.01 (1.00–1.01, *p* = 0.130)	
TBIL	Mean ± SD	27.8 ± 42.4	1.00 (1.00–1.01, *p* = 0.406)	
ALB	Mean ± SD	25.3 ± 6.4	1.04 (1.00–1.09, *p* = 0.078)	
CREA	Mean ± SD	201.3 ± 178.1	1.00 (1.00–1.00, *p* = 0.472)	
K	Mean ± SD	4.7 ± 0.9	1.18 (0.89–1.58, *p* = 0.257)	
Na	Mean ± SD	145.4 ± 9.3	1.03 (1.00–1.06, *p* = 0.032)	
Lac	Mean ± SD	6.3 ± 4.9	1.13 (1.08–1.19, *p* < 0.001)	1.10 (1.02–1.17, *p* = 0.008)
Vasopressors	No	90 (38.6%)		
	Yes	143 (61.4%)	6.97 (2.48–19.59, *p* < 0.001)	5.16 (1.73–15.38, *p* = 0.003)
CRRT	No	213 (91.4%)		
	Yes	20 (8.6%)	1.24 (0.44–3.48, *p* = 0.685)	
SOFA	Mean ± SD	10.2 ± 4.0	1.15 (1.07–1.24, *p* < 0.001)	0.93 (0.82–1.04, *p* = 0.193)
HCT	High	197 (84.5%)		
	Low	36 (15.5%)	2.75 (1.40–5.41, *p* = 0.003)	2.16 (1.04–4.52, *p* = 0.040)

COPD, chronic obstructive pulmonary disease; CHD, coronary heart disease; CKD, chronic kidney disease; MAP, mean arterial pressure; HGB, hemoglobin; NEUT, neutrophil; PLT, platelet; PCT, procalcitonin; TBIL, total bilirubin; ALB, albumin; CREA, creatinine; HCT, hematocrit.

## Discussion

In an international study, sepsis was identified as an underlying cause in approximately 75% of patients with acute respiratory distress syndrome (ARDS), with an estimated 210,000 cases of sepsis-induced ARDS occurring annually in the US alone (15, 16). Sepsis-induced acute respiratory distress syndrome (ARDS) has been shown to have a higher mortality rate than ARDS from other causes (16). Mechanical ventilation (MV) is the cornerstone of supportive care for this severe condition, and a substantial proportion of sepsis patients require MV—13.5% overall and over half within the first 24 h of presentation (17). Given the high prevalence and prognostic relevance of acute respiratory distress syndrome (ARDS) among patients with sepsis, readily accessible prognostic tools are imperative for this high-risk subgroup. A mounting body of evidence has revealed a correlation between hematocrit (HCT) and all-cause mortality across a range of conditions, including coronary heart disease, heart failure, end-stage renal disease, cancer, and inflammatory states (18). However, the specific role of HCT in mechanically ventilated sepsis patients remains poorly characterized. Accordingly, the objective of this study was to evaluate the association between HCT and 28-day mortality in sepsis patients receiving MV. In this multicenter study, an exploratory HCT cutoff of 26.65% was identified, and it was found that low HCT was associated with a 2.16-fold increased risk of 28-day mortality. These findings suggest that HCT may serve as a useful prognostic indicator specifically in sepsis patients receiving MV, addressing a distinct gap in the existing literature.

The well-established prognostic value of HCT in critical illness suggests that its pathophysiological role in sepsis merits further elaboration. Hematocrit (HCT), defined as the volumetric proportion of red blood cells (RBC), is intrinsically linked to hemoglobin (Hb) concentration. These two parameters are fundamental for assessing anemia and oxygen-carrying capacity (19, 20). Anemia is a prevalent complication in sepsis, driven by multifactorial mechanisms. Systemic inflammation directly suppresses erythropoiesis, while circulating bacteria and complement activation damage existing erythrocytes, leading to their premature clearance or intravascular hemolysis (21). The resultant decline in HCT and Hb has been demonstrated to impair systemic oxygen delivery, potentially inducing tissue hypoxia and exacerbating microcirculatory dysfunction. This, in turn, has been shown to promote the progression of septic shock. Consequently, HCT levels are theorized to influence clinical outcomes critically—a premise that is supported by evidence linking low HCT to increased 30-day mortality in sepsis, suggesting its prognostic relevance (9).

In consideration of this pathophysiological support, we sought to clarify the relationship between HCT and hemoglobin as prognostic biomarkers. A systematic review and meta-analysis established hemoglobin (Hb) as a reliable prognostic marker in sepsis, highlighting the critical relationship between oxygen-carrying capacity and survival (22). The present findings extend this principle by demonstrating that HCT also exhibits a statistically significant association with 28-day mortality in mechanically ventilated sepsis patients. Although Hb and HCT are inherently associated, the unique pathophysiology of our cohort may render HCT particularly pertinent. These patients frequently exhibit substantial fluid shifts due to aggressive fluid resuscitation and capillary leakage (23, 24). In this context, HCT, when expressed as a volumetric percentage, can function as a more dynamic integrator of both red cell mass and plasma volume. This enhanced integration provides a more comprehensive reflection of oxygen delivery in the context of a dynamically changing intravascular space. However, it is equally important to consider the possibility that low HCT in this population may primarily reflect underlying disease severity rather than constituting a direct causal driver of mortality. In critically ill septic patients, HCT levels are influenced by multiple factors beyond the disease itself, including intravenous fluid resuscitation (hemodilution), capillary leak, bleeding events, red blood cell transfusions, renal dysfunction, and preexisting chronic anemia. These factors are closely intertwined with illness severity and may themselves affect prognosis, making it challenging to isolate the independent contribution of HCT to mortality and warranting caution in causal interpretation.

Several limitations of this study warrant consideration. First, the observational design precludes causal inference. Although we adjusted for measured confounders, residual confounding from unmeasured variables remains a concern, particularly due to missing data on key factors influencing HCT and prognosis, including transfusions, fluid balance, bleeding events, surgical interventions, baseline anemia, and ventilator settings. While SOFA and lactate were included as proxies of illness severity, the absence of these variables limits complete adjustment, potentially biasing estimates. Thus, our findings should be interpreted as associative rather than causal. Second, the modest event count (*n* = 40) constrained multivariable modeling under the events-per-variable rule, necessitating the exclusion of several clinically relevant covariates (e.g., age, malignancy, platelet count, renal function, and albumin) and predisposing the model to overfitting. This instability was corroborated by bootstrap validation, which yielded a 95% confidence interval for the HCT hazard ratio ranging from 0.858 to 6.579—crossing unity. Moreover, the low-HCT subgroup comprised only 36 patients (12 deaths), further contributing to broad confidence intervals around the adjusted hazard ratio and limited statistical precision.

It is acknowledged that the exploratory candidate threshold of 26.65% was derived from the current dataset using the Youden index. However, this approach carries inherent risks of overfitting and limits the generalizability to other populations. To address this concern, an internal bootstrap validation was performed, which demonstrated that the original estimate fell within the interquartile range of the bootstrap-derived cutoffs (26.65–35.60%), suggesting reasonable internal consistency in this cohort. However, it is imperative to acknowledge that this internal stability does not supersede the necessity for external validation. Therefore, it is imperative to emphasize that this threshold should be regarded as a preliminary exploratory finding and should not be interpreted as a clinically established action point. In summary, while low HCT was associated with increased mortality in this cohort, with 26.65% identified as a potential reference point, the near-chance discrimination (AUC ≈ 0.537) precludes any strong claim for HCT as a standalone prognostic biomarker. Despite its routine measurement and affordability, its role should be considered adjunctive. Its practical usefulness for risk stratification remains uncertain without further validation and integration with other clinical variables.

## Conclusion

In this cohort of mechanically ventilated sepsis patients, low HCT was associated with an increased risk of 28-day mortality. In light of the restricted subgroup size and substantial confidence intervals, this finding should be regarded as exploratory and hypothesis-generating, necessitating validation in larger, well-powered studies. Despite the widespread availability and cost-effectiveness of HCT, its limited discriminative capacity hinders its application as a standalone risk stratification tool. Instead, it may function as an ancillary parameter that, when integrated with other clinical and laboratory markers, could enable early risk assessment. Further studies are needed to validate its incremental prognostic value in this population.

## Data Availability

The datasets analyzed during the current study are available from the corresponding author on reasonable request.
