# Supplementary material for: Hematocrit and 28-day mortality in mechanically ventilated septic patients: a prospective observational study
Source: Front Med (Lausanne). 2026 Aug 26;13:1890758. doi: 10.3389/fmed.2026.1890758 (PMC13553920; doi:10.3389/fmed.2026.1890758)
Supplement: Supplementary file 1 [file Table_1.DOCX]

Appendix 1: Variance Inflation Factor Among Candidate Predictors
